# Supplementary material for: PathwayPilot: A User-Friendly Tool for Visualizing and Navigating Metabolic Pathways
Source: Mol Cell Proteomics. 2025 Jan 27;24(3):100918. doi: 10.1016/j.mcpro.2025.100918 (PMC11903815; doi:10.1016/j.mcpro.2025.100918)
Supplement: Supplemental data [file mmc3.docx]

Supplemental data

PathwayPilot: A User-Friendly Tool for Visualizing and Navigating Metabolic Pathways

Tibo Vande Moortele, Pieter Verschaffelt, Qingyao Huang, Nadezhda T. Doncheva, Tanja Holstein, Caroline Jachmann, Peter Dawyndt, Lennart Martens, Bart Mesuere*, Tim Van Den Bossche

* corresponding author: [unipept@ugent.be](mailto:unipept@ugent.be)

# Separate files

- **S1 - peptides.xlsx**: A full list of peptides that match within one of the analysed pathways in Tanca et al.
- **S2 - abundances.xlsx**: A detailed overview of all abundance results for the case study.
